# MIP-1δ Activates NFATc1 and Enhances Osteoclastogenesis: Involvement of Both PLCγ2 and NFκB Signaling

**DOI:** 10.1371/journal.pone.0040799

**Published:** 2012-07-09

**Authors:** Kristy L. Weber, Michele Doucet, Adam Shaner, Nigel Hsu, David Huang, Jenna Fogel, Scott L. Kominsky

**Affiliations:** Department of Orthopaedic Surgery, Johns Hopkins University School of Medicine, Baltimore, Maryland, United States of America; University of Leuven, Rega Institute, Belgium

## Abstract

Pathological bone resorption is a source of significant morbidity in diseases affecting the skeleton such as rheumatoid arthritis, periodontitis, and cancer metastasis to bone. Evidence indicates that elevated levels of inflammatory mediators such as IL-1, IL-6, and TNF-α play a role in this process by promoting the formation of bone-resorbing osteoclasts. Additionally, current studies have identified inflammatory chemokines of the macrophage inflammatory protein (MIP) family as potential mediators of pathological bone resorption, where both MIP-1α and -3α have been shown to enhance osteoclast (OCL) development. In this study we provide evidence that MIP-1δ, whose expression is associated with renal cell carcinoma bone metastasis and rheumatoid arthritis, enhances OCL formation in vitro via a direct effect on OCL precursors. Consistent with this ability, exposure of OCL precursors to MIP-1δ resulted in the activation of PLCγ2 and NF-κB, two signaling pathways known to regulate OCL differentiation. Moreover, MIP-1δ induced expression and nuclear translocation of NFATc1, a master regulator of osteoclastogenesis, which was dependent on activation of both the PLCγ2 and NFκB signaling pathways. Lastly, consistent with in vitro studies, in vivo administration of MIP-1δ significantly increased OCL number and resorption area as determined using a murine calvarial bone resorption model. Taken together, these data highlight the potential of MIP-1δ as a mediator of pathological bone resorption and provide insight into the molecular mechanism through which MIP-1δ enhances osteoclastogenesis.

## Introduction

Pathological bone resorption occurs in skeletal diseases such as rheumatoid arthritis, periodontitis, and cancer, resulting in substantial bone pain and loss of function. In the case of rheumatoid arthritis and periodontitis, bone loss occurs following chronic inflammation. Inflammatory mediators such as interleukin (IL)-1, IL-6, and tumor necrosis factor (TNF)-α have been shown to elevate levels of the osteoclastogenic cytokine, receptor activator of nuclear factor kappa-B ligand (RANKL), enhancing the development of bone resorbing osteoclasts (OCL) [1], thereby disrupting the delicate balance of bone resorption and formation. Evidence also supports a role for inflammatory mediators (eg. IL-3, IL-6, and IL-8) in the OCL-mediated bone resorption observed in metastatic breast cancer and multiple myeloma [2], [3], [4].

Recent studies indicate that inflammatory chemokines of the macrophage inflammatory protein (MIP) family may also play a role in mediating pathological bone resorption. Currently, the MIP family consists of six members: MIP-1α, MIP-1β, MIP-1δ, MIP-1γ, MIP-3α, and MIP-3β. MIP-3α, whose expression is increased in bone biopsies from rheumatoid arthritis patients, has been shown to enhance OCL development by stimulating OCL precursor proliferation [5]. Similarly, it has also been detected in periodontitis where elevated expression was positively correlated with disease status [6], [7], [8]. Elevated levels of another family member, MIP-1α, were reported in bone marrow of multiple myeloma patients as compared to healthy adults [9]. Further studies indicate that MIP-1α is able to stimulate OCL development [10], while inhibition of MIP-1α significantly reduces bone destruction in a mouse model of multiple myeloma [11].

In line with these findings, we recently found levels of another MIP family member, MIP-1δ (CCL15), to be significantly elevated in human renal cell carcinoma bone metastasis (RBM) tissues relative to bone marrow from healthy adults [12]. Further, consistent with the osteolytic nature of RBM, we provided in vitro evidence that MIP-1δ stimulates chemotaxis of OCL progenitors and enhances OCL differentiation in response to RANKL. Here, we demonstrate the ability of MIP-1δ to directly enhance the differentiation of OCL precursors in vitro, elucidate its effect on the signaling pathways and transcription factors regulating osteoclastogenesis, and provide the first evidence that MIP-1δ can stimulate osteoclastogenesis and bone resorption in vivo, highlighting its potential as a mediator of pathological bone loss.

## Results and Discussion

### MIP-1δ Enhances Osteoclastogenesis in vitro

**Figure 1:**
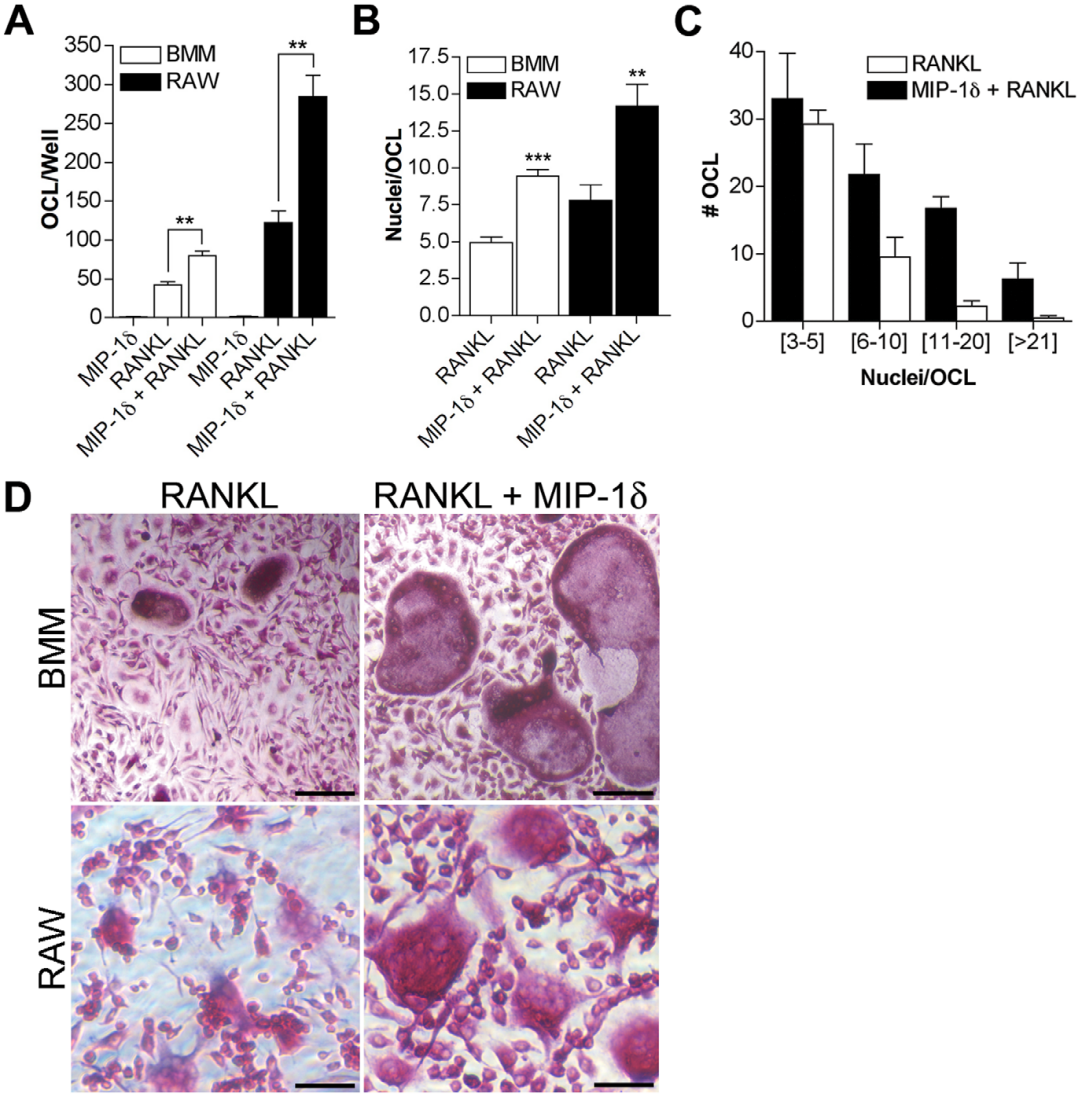
MIP-1δ enhances RANKL-induced osteoclastogenesis in vitro. BMM or RAW 264.7 cells were treated with MIP-1δ (0.1 pg/ml) and RANKL (5 ng/ml) individually or in combination for 7 days. (**A**) OCL formation was assessed by counting the total number of OCL per well under light microscopy (**D**, 200X Magnification; Scale bars, 50 µm**)**. (**B, C**) Fusion index was determined by counting the number of nuclei per OCL in 15–20 random fields and is reported as (B) the average number of nuclei per OCL and (C) the number of OCL containing a specified range of nuclei per cell (BMM shown, similar results were observed for RAW 264.7). Data are representative of three independent experiments performed in quadruplicate, expressed as the mean (n  = 12) ± s.e.m, and were analyzed by unpaired, two-tailed Student’s t-test. **, *p*<0.01; ***, *p*<0.001.

Previously, we reported the first evidence that MIP-1δ enhances RANKL-mediated OCL differentiation in vitro using murine bone marrow mononuclear cells (BM-MNC) [12]. Since BM-MNC is a heterogeneous population containing a minor fraction of OCL progenitors, it was unclear whether MIP-1δ affected OCL differentiation via a direct effect on OCL progenitors or through indirect effects on other cells within the population (eg. marrow stromal cells). Thus, we examined the ability of MIP-1δ to promote OCL differentiation in vitro using macrophage colony-stimulating factor (M-CSF)-dependent bone marrow macrophages (BMM), a refined population of committed OCL precursors. Consistent with our previous findings [12], while insufficient to stimulate OCL differentiation alone, MIP-1δ significantly enhanced OCL differentiation in response to RANKL, evidenced by increased numbers of TRAP-positive multinucleated cells (Fig. 1A). In addition, OCL formed in the presence of MIP-1δ exhibited a significantly higher fusion index (Fig. 1B) compared to those treated with RANKL alone, highlighted by a greater than 7-fold increase in OCL containing more than 11 nuclei per cell (Fig. 1C, D). Notably, similar results were observed using the macrophage cell line RAW 264.7 (Fig. 1A, B, D), indicating that our findings using BMM were not likely affected by any minor stromal elements remaining within the BMM population. Taken together, these data suggest that MIP-1δ enhances OCL formation via a direct effect on OCL precursors.

### MIP-1δ Activates Key Pathways Regulating Osteoclastogenesis

**Figure 2:**
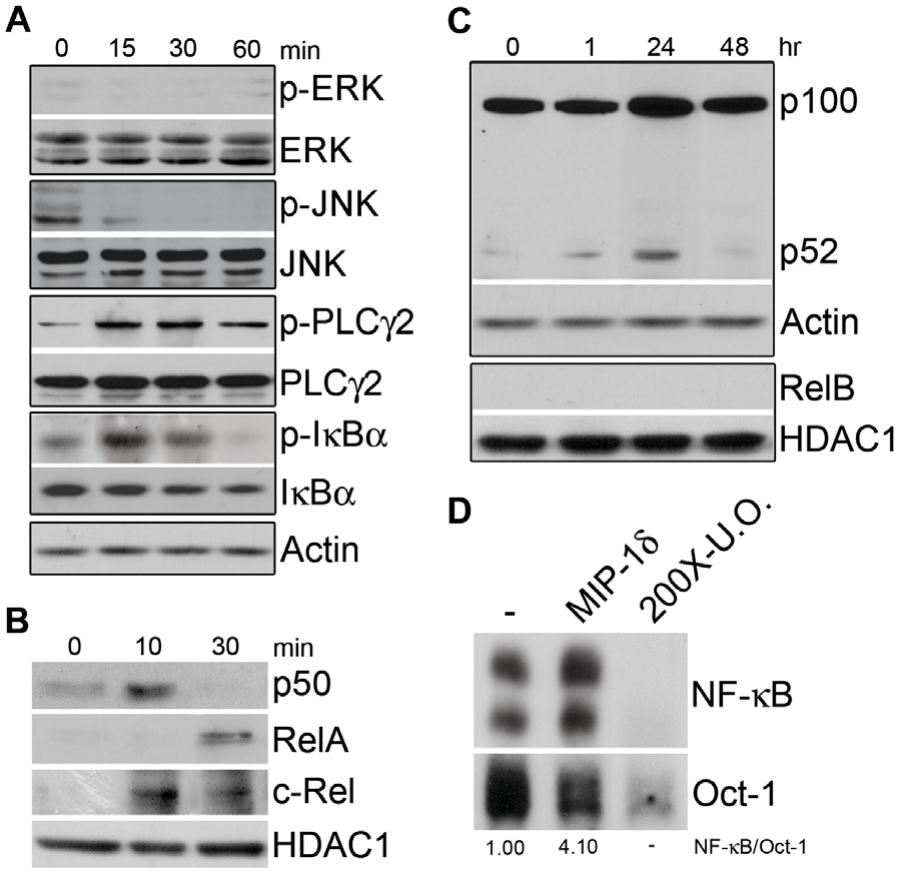
MIP-1δ activates PLCγ2 and NF-κB signaling pathways in OCL progenitors. BMM were treated with or without MIP-1δ (0.1 pg/ml) and the effect on the ERK, JNK, PLCγ2, and NF-κB signaling pathways was examined by Western analysis. (**A**) The effect of MIP-1δ on the levels of total and phospho (p)-ERK, JNK, PLCγ2, and IκBα was examined using equal amounts of protein from total cell lysates. (**B**) Nuclear translocation of p50, RelA, c-Rel, and (**C**) RelB following MIP-1δ treatment was determined using equal amounts of protein from nuclear cell lysates. (**C**) Processing of p100 to p52 following MIP-1δ treatment was determined using equal amounts of protein from total cell lysates. (**D**) Activation of NF-κB was determined 30 minutes following treatment with MIP-1δ by non-radioactive EMSA analysis of nuclear cell lysates using NF-κB oligonucleotides. Oct-1 served as a loading control and 200-fold excess unlabeled oligonucleotide (200X-U.O.) served as a competitive control. Densitometry values are provided below respective lanes for ease of interpretation. Data are representative of at least two independent experiments.

Prior studies identified several signaling pathways through which OCL differentiation is promoted, including ERK [13], JNK [14], PLCγ2 [15], and NF-κB [16]. To investigate the mechanism whereby MIP-1δ enhances OCL differentiation, we first determined its ability to activate each of these signaling pathways in OCL precursors. Although exposure of BMM to MIP-1δ did not induce phosphorylation of ERK or JNK, increased phosphorylation of PLCγ2 was observed, indicating activation of PLCγ2 signaling (Fig. 2A). In addition, MIP-1δ demonstrated an effect on both the canonical and non-canonical NF-κB signaling pathways. While activation of the canonical NF-κB pathway depends on phosphorylation and subsequent degradation of IκBs, largely IκBα, the non-canonical pathway depends on processing of p100 to p52 [17]. In addition, p50 traditionally partners with RelA or c-Rel in canonical signaling, while p52 partners with RelB in non-canonical signaling. Following exposure of BMM to MIP-1δ, we observed phosphorylation and degradation of IκBα (Fig. 2A). Further, analysis of nuclear extracts revealed the translocation of p50 and its binding partners RelA and c-rel to the nucleus, indicating activation of canonical NF-κB signaling (Fig. *2*B). It is also interesting to note that the kinetics of p50 and c-Rel nuclear translocation following stimulation with MIP-1δ appear shorter than that of RelA. While this apparent shift in kinetics may be due to a number of events (*eg.* differences in NF-κB subunit degradation kinetics, nuclear translocation of p50 homodimers, etc.), this data suggests that MIP-1δ may have a unique effect on NF-κB signaling, which may be of interest in subsequent studies. Examination of non-canonical NF-κB signaling revealed processing of p100 to p52 following stimulation with MIP-1δ, however, nuclear translocation of the p52 binding partner RelB was not detected (Fig. 2C), suggesting that MIP-1δ may favor activation of the canonical pathway**.** Lastly, using an oligonucleotide probe containing an NF-κB binding site, we observed increased NF-κB activity in response to MIP-1δ stimulation of BMM by EMSA (Fig. 2D). Collectively, these data demonstrate the ability of MIP-1δ to activate both PLCγ2 and NF-κB signaling in OCL precursors, each having the potential to contribute to the effects of MIP-1δ on OCL formation.

### MIP-1δ Induces Critical Regulators of Osteoclastogenesis via activation of PLCγ2 and NF-κB

**Figure 3:**
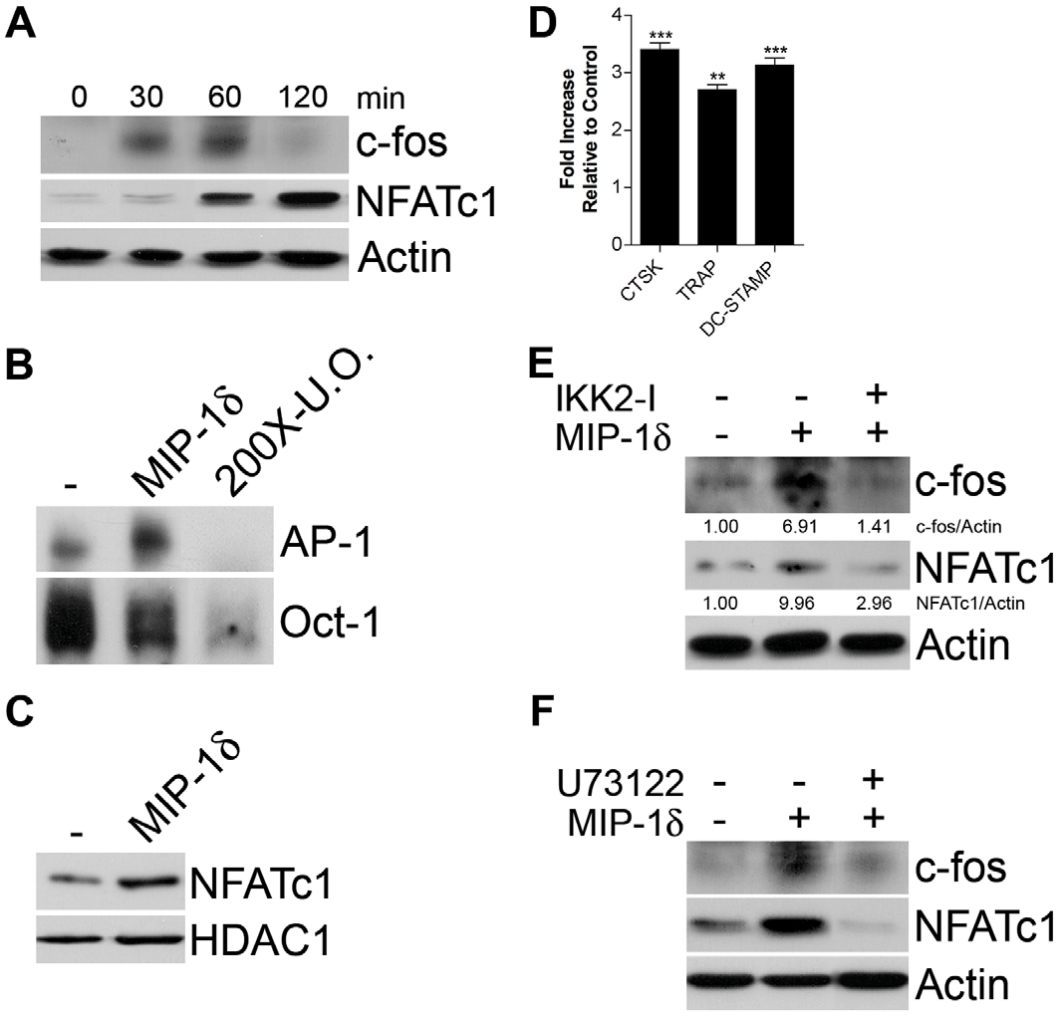
MIP-1δ induces expression and activity of c-fos and NFATc1 via stimulation of the PLCγ2 and NF-κB signaling pathways. BMM were treated with or without MIP-1δ (0.1 pg/ml). (A) c-fos and NFATc1 protein expression were determined by Western analysis performed on equal amounts of protein from total cell lysates. (B) Activation of AP-1 was determined 30 minutes following treatment with MIP-1δ by non-radioactive EMSA analysis of nuclear cell lysates using AP-1 oligonucleotides. Oct-1 served as a loading control and 200-fold excess unlabeled oligonucleotide (200X-U.O.) served as a competitive control. (C) NFATc1 protein expression was determined 60 minutes following treatment with MIP-1δ by Western analysis performed on equal amounts of protein from nuclear cell lysates. (D) CTSK, TRAP, and DC-STAMP mRNA expression were determined by qRT-PCR 24 hours following treatment with MIP-1δ. Data are representative of three independent experiments performed in triplicate, expressed as the mean (n  = 9) ± s.e.m, and were analyzed by unpaired, two-tailed Student’s t-test. *p*<0.01; ***, *p*<0.001. (E, F) BMM were treated with MIP-1δ (0.1 pg/ml) alone or in combination with the (E) NF-κB inhibitor IKK2 inhibitor VIII (IKK2-I) or the (F) PLC inhibitor U73122 and protein expression was determined 60 minutes (c-fos, NFATc1) or 30 minutes (IκBα, phospho-PLCγ2) following MIP-1δ treatment by Western analysis performed on equal amounts of protein from total cell lysates. (E) Densitometry values are provided below respective lanes for ease of interpretation. Data are representative of at least two independent experiments.

Recent studies suggest that the importance of the PLCγ2 and NF-κB signaling pathways to osteoclastogenesis lies in their ability to stimulate expression and nuclear translocation of the transcription factor NFATc1, considered the “master regulator of OCL differentiation” [18], [19]. NFATc1 auto-regulates its promoter together with c-fos, a component of the dimeric transcription factor AP-1, and controls expression of genes critical to OCL differentiation and function [20]. Because MIP-1δ activated both the PLCγ2 and NF-κB signaling pathways, we next examined its effect on expression and activity of c-fos and NFATc1. Treatment of BMM with MIP-1δ significantly increased expression of c-fos (Fig. 3A) and, correspondingly, increased activation of AP-1 as determined by EMSA analysis (Fig. 3B). Moreover, similar to its effect on c-fos expression, MIP-1δ significantly increased expression (Fig. 3A) and translocation of NFATc1 to the nucleus (Fig. 3C). Correspondingly, increased mRNA expression of the NFATc1 downstream targets cathepsin K (CTSK), tartrate-resistant acid phosphatase (TRAP), and dendritic cell-specific transmembrane protein (DC-STAMP) was also observed (Fig. 3D). To determine the contribution of the NF-κB and PLCγ2 signaling pathways to MIP-1δ-induced expression of c-fos and NFATc1 we examined the effect of MIP-1δ in the presence of chemical inhibitors of each pathway. Inhibition of both canonical and non-canonical NF-κB signaling using an IKK-2 inhibitor prevented c-fos and NFATc1 induction by MIP-1δ (Fig. 3E). Similarly, exposure of BMM to the PLC inhibitor U73122 also inhibited the ability of MIP-1δ to induce c-fos and NFATc1 expression (Fig. 3F). Taken together, these data indicate that MIP-1δ induces the expression and activation of c-fos and NFATc1, critical for OCL differentiation, involving stimulation of both the PLCγ2 and NF-κB pathways.

It is interesting to note that although able to induce NFATc1 expression, MIP-1δ was unable to induce OCL formation in the absence of RANKL ([12], data not shown). While seemingly incongruous, NFATc1 is reported to be necessary for OCL differentiation, yet insufficient in the absence of RANKL [21], indicating an additional effect of RANKL beyond the induction of NFATc1. Intriguingly, while both MIP-1δ and RANKL activate NF-κB, MIP-1δ appears to incompletely stimulate the non-canonical pathway, evidenced by the lack of RelB nuclear translocation (Fig. 2C). Given the importance of the non-canonical pathway for OCL differentiation [22], it is tempting to speculate that the limited ability of MIP-1δ to stimulate this pathway contributes to its failure to induce OCL differentiation independently of RANKL. Despite a limited capacity to stimulate non-canonical NF-κB signaling, MIP-1δ robustly activated the canonical pathway as evidenced by the nuclear translocation of RelA and c-Rel (Fig. 2B). Notably, canonical NF-κB signaling through induction of RelA has been reported to promote survival of OCL precursors, translating to increased OCL formation [23]. Thus, although unable to induce OCL differentiation alone, MIP-1δ may enhance OCL formation not only by promoting differentiation via NFATc1 induction, but also by promoting survival of OCL progenitors via activation of RelA.

### MIP-1δ Enhances Osteoclastogenesis and Bone Resorption in vivo

**Figure 4:**
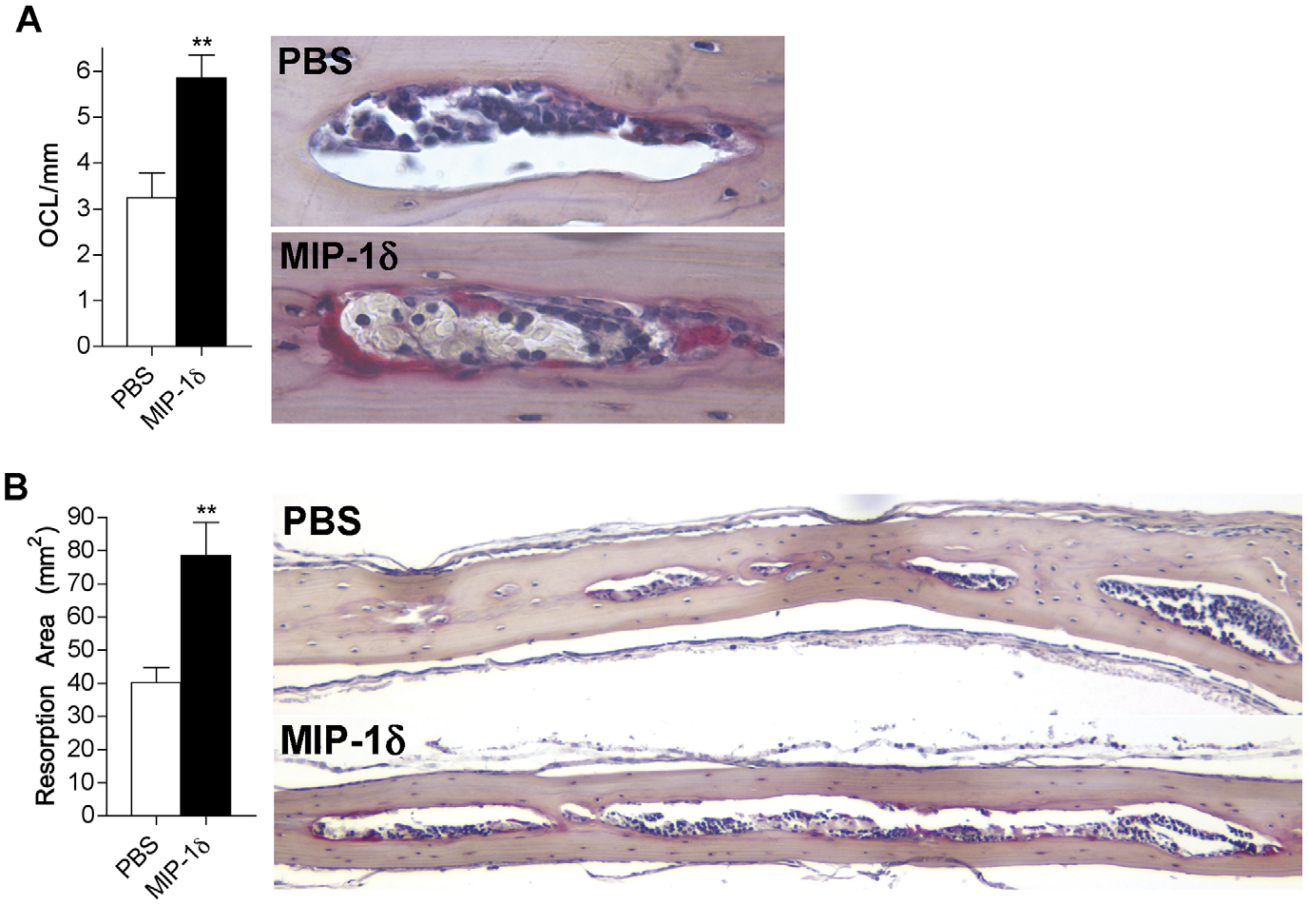
MIP-1δ promotes osteoclastogenesis and bone resorption in vivo. Mice (5/group) were administered MIP-1δ (1 ng/ml) or PBS subcutaneously over the calvariae twice each day for 5 days. Calvariae were removed and paraffin-embedded sections were generated for histomorphometric analysis. (A) OCL were identified by TRAP staining, counted under light microscopy (right, 400X), and reported relative to mm of marrow cavity surface (left). (B) Resorption area (left) was determined by measuring the total area occupied by marrow cavities within each calvarial section (right, 100X). Data are representative of two independent experiments, expressed as the mean (n  = 10) ± s.e.m, and were analyzed by unpaired, two-tailed Student’s t-test. **, *p*<0.01.

Although MIP-1δ levels are elevated in several conditions causing pathological bone loss [12], [24] and we have demonstrated its ability to enhance OCL differentiation in vitro, the capacity of MIP-1δ to induce osteolysis in vivo has not been investigated. To determine whether MIP-1δ affects osteoclastogenesis and bone resorption in vivo, we utilized a murine model wherein MIP-1δ or PBS was injected subcutaneously over the calvariae. Consistent with the ability of MIP-1δ to enhance OCL differentiation in vitro, calvariae from mice administered MIP-1δ showed a significant increase in OCL number and resorption area relative to that observed in mice administered PBS (Fig. 4). These data indicate that MIP-1δ is able to increase osteoclastogenesis and bone resorption, underscoring its potential to contribute to pathological bone loss.

While we have demonstrated the ability of MIP-1δ to directly influence OCL precursors in vitro, it is possible that MIP-1δ promotes OCL formation and bone resorption in vivo via additional mechanisms. Interestingly, expression of the receptors for MIP-1δ, CCR1 and CCR3, is increased in mature OCL relative to OCL precursors [12]. Therefore it is conceivable that MIP-1δ exerts effects on mature OCL in addition to OCL precursors, possibly influencing survival and activity. In addition to effects on bone-resorbing OCL, it is also possible that MIP-1δ modulates the activity of bone-forming osteoblasts (OB). OB play a major role in OCL differentiation by producing RANKL, and its decoy receptor, osteoprotegerin (OPG). Since OB also express the receptors for MIP-δ (S.L.K., unpublished observations/data), it may indirectly promote OCL formation and bone loss in vivo by influencing the production of RANKL and OPG by OB as well as by regulating OB survival and activity. Thus, in future studies it will be important to examine the effects of MIP-1δ on mature OCL as well as pre and mature OB.

Collectively, these data highlight the potential of MIP-1δ to contribute to pathologic bone resorption associated with skeletal diseases such as rheumatoid arthritis and cancer metastasis to bone, where MIP-1δ levels are elevated [12], [25]. This work is timely as small molecule antagonists against CCRs are being developed and evaluated in clinical trials [26]. While this approach is promising, caution must be taken as many CCRs, including CCR1 and CCR3, are widely expressed in the body and serve as receptors for multiple ligands raising the potential for toxicity. Further investigation into the molecular mechanisms through which MIP-1δ influences OCL formation and bone resorption in vivo are warranted and may lead to the discovery of more specific methods of inhibiting its effects.

## Materials and Methods

### Ethics Statement

All experiments were carried out in accordance with the National Research Council’s “Guide to the Care and Use of Laboratory Animals”. Animal use was approved by the Johns Hopkins Animal Care and Use Committee, animal welfare assurance #A3272-01, protocol #MO07M149.

### Generation of Bone Marrow Macrophages

Bone marrow was isolated from the tibiae, femur, and humerus of 4–8 week old NIH Swiss mice. The ends of each bone were removed and the marrow collected by flushing the bone with αMEM containing 15% FBS and 1% penicillin and streptomycin (P/S) using a 27 gauge needle. Cells were washed and re-suspended in complete media (αMEM +10% FBS +1% P/S) containing 5 ng/ml M-CSF (R&D Systems, Minneapolis, MN) and incubated in a 100 mm dish overnight. Non-adherent cells were then collected, strained through 70 µm nylon mesh, and treated with 50 ng/ml M-CSF for 3 days to generate M-CSF-dependent bone marrow macrophages (BMM).

### Osteoclast Differentiation Assay

BMM were cultured in complete media containing M-CSF (20 ng/ml) and RANKL (5 ng/ml) (R&D Systems) in the presence or absence of full-length recombinant human MIP-1δ (0.1 pg/ml) (R&D Systems). 50% of the culture media was replaced with fresh complete media containing cytokines as appropriate every 3 days. After 7 days, the development of mature osteoclasts (OCL) was determined by positive tartrate-resistant acid phosphatase (TRAP) staining (bright red color) and the presence of ≥3 nuclei under light microscopy. To determine the fusion index, the number of nuclei per OCL was counted in 15 to 20 random fields at 100X magnification.

### In vivo Calvarial Bone Resorption

Following a previously established procedure [27], recombinant human MIP-1δ (1 ng) or PBS was injected in a volume of 50 µl into the subcutaneous tissue overlying the right side of the parietal bones of calvariae in female 6-week-old CD-1 mice twice each day for 5 days. Four days following the last injection, calvariae were harvested, fixed in formalin for 24 hours, and decalcified in 10% EDTA, pH 7.4 for 48 hours. Paraffin-embedded sections (5 µm) were generated laterally from the sagittal suture at 100 µm intervals. A minimum of three sections were stained with orange G and TRAP, and light microscopic images were acquired. The number of OCL/mm of marrow cavity surface and total resorption area (the total area occupied by marrow cavities) between the coronal and lamboid sutures was measured using MetaMorph image analysis software (Universal Imaging Corp., Sunnyvale, CA).

### Quantitative (q)RT-PCR

Total RNA was extracted using Trizol (Invitrogen) and cDNA was generated by reverse transcription. 25 µl reactions contained 1X SYBR Green Reaction Mix (Applied Biosystems), 1 µl cDNA, and 100 nm of each primer: CTSK (sense) 5′-CTCGGCGTTTAATTTGGGAGA-3′, (antisense) 5′-TCCAGGTTATGGGCAGAGATT-3′; TRAP (sense) 5′-GTGCTGCTGGGCCTACAAAT-3′, (antisense) 5′-TTCTGGCGATCTCTTTGGCAT-3′; DC-STAMP (sense) 5′-TGGAAGTTCACTTGAAACTACGTG-3′ (anti-sense) 5′-CTCGGTTTCCCGTCAGCCTCTCTC-3′; GAPDH (sense) 5′- AACTTTGGCATTGTGGAAGG-3′, (antisense) 5′-ACACATTGGGGGTAGGAACA-3′. qRT-PCR parameters were: 1 cycle (95°C for 3 minutes) and 40 cycles (95°C for 30 seconds, 61.9°C for 30 seconds, and 72°C for 45 seconds). Amplification of GAPDH was used as an internal control. Relative expression between samples was calculated by the comparative C_T_ method.

### Western and EMSA Analysis

BMM were cultured in media without serum or M-CSF for 6 hours and subsequently treated with MIP-1δ (0.1 pg/ml) for time periods indicated in Figs. 2, 3. For some experiments, BMM were cultured in the presence of chemical inhibitors specific to the NF-κB (IKK-2 inhibitor VIII, EMD Chemicals, Gibbstown, NJ) and PLCγ2 (U73122, Cayman Chemical, Ann Arbor, MI) pathways as indicated in Fig. 3E,F. IKK-2 inhibitor VIII and U73122 were used at 20 µM and 5 µM, respectively, consistent with that utilized in similar studies [15], [28], [29]. Protein expression/phosphorylation was detected as described [12]. For examination of nuclear protein levels and EMSA analysis, nuclear cell lysates were generated using NE-PER Nuclear and Cytoplasmic Extraction Reagent (Thermo Scientific, Rockford, IL). Antibodies were obtained from Cell Signaling (Danvers, MA) unless otherwise indicated: ERK, p-ERK, JNK, p-JNK, PLCγ2, p-PLCγ2, IκBα, p-IκBα, p50, p52, RelA, c-Rel, RelB, c-fos (Santa Cruz Biotechnology, Santa Cruz, CA), and NFATc1 (Santa Cruz Biotechnology). β-actin (Sigma-Aldrich, St. Louis, MO) and HDAC1 served as loading controls.

Non-radioactive EMSA was performed on nuclear extracts following the manufacturer’s instructions (Thermo Scientific) using AP1 (5′-Biotin-ACGCTTGATGACTCAGCCGGAAT-3′ and 5′-Biotin-ATTCCGGCTGAGTCATCAAGC-3′) and NF-κB (5′-Biotin-AAGTTGAGGGGACTTTCCCAGGCT-3′ and 5′-Biotin-AGCCTGGGAAAGTCCCCTCAACTT-3′) oligonucleotides. Oct-1 oligonucleotide (5'-Biotin-TGTCGAATGCAAATCACTAGAA-3') was used as a loading control and a 200-fold excess of non-biotinylated oligonucleotides was used as a competitive control for binding specificity. NF-κB and AP-1 activity were evaluated concurrently, thus the same Oct-1 bands appear in Figs. 2D, 3B.
